# Efficiency Analysis of Direct Video-Assisted Thoracoscopic Surgery in Elderly Patients with Blunt Traumatic Hemothorax without an Initial Thoracostomy

**DOI:** 10.1155/2016/3741426

**Published:** 2016-04-14

**Authors:** Wen-Yen Huang, I-Yin Lu, Chyan Yang, Yi-Pin Chou, Hsing-Lin Lin

**Affiliations:** ^1^Division of Trauma, Department of Emergency, Kaohsiung Veterans General Hospital, Kaohsiung 807, Taiwan; ^2^Institute of Business and Management, National Chiao Tung University, Hsinchu 300, Taiwan; ^3^Research Center for Industry of Human Ecology, Chang Gung University of Science and Technology, Kweishan, Taoyuan 330, Taiwan; ^4^Emergency Department, Fooyin University Hospital, Pingtung County 928, Taiwan; ^5^Department of Medical Technology, Fooyin University, Kaohsiung 928, Taiwan; ^6^Department of Nursing, Tajen University, Yanpu Township, Pingtung County 928, Taiwan; ^7^Department of Emergency Medicine, Kaohsiung Medical University Hospital, Kaohsiung Medical University, Kaohsiung 807, Taiwan; ^8^Department of Emergency Medicine, Faculty of Medicine, College of Medicine, Kaohsiung Medical University, Kaohsiung 807, Taiwan

## Abstract

Hemothorax is common in elderly patients following blunt chest trauma. Traditionally, tube thoracostomy is the first choice for managing this complication. The goal of this study was to determine the benefits of this approach in elderly patients with and without an initial tube thoracostomy. Seventy-eight patients aged >65 years with blunt chest trauma and stable vital signs were included. All of them had more than 300 mL of hemothorax, indicating that a tube thoracostomy was necessary. The basic demographic data and clinical outcomes of patients with hemothorax who underwent direct video-assisted thoracoscopic surgery without a tube thoracostomy were compared with those who received an initial tube thoracostomy. Patients who did not receive a thoracostomy had lower posttrauma infection rates (28.6% versus 56.3%, *P* = 0.061) and a significantly shorter length of stay in the intensive care unit (3.13 versus 8.27, *P* = 0.029) and in the hospital (15.93 versus 23.17, *P* = 0.01) compared with those who received a thoracostomy. The clinical outcomes in the patients who received direct VATS were more favorable compared with those of the patients who did not receive direct VATS.

## 1. Introduction

Trauma is among the leading causes of death in elderly patients. Elderly adults tend to have chronic disorders such as hypertension, chronic obstructive pulmonary disease, diabetes, and coronary artery disease more frequently than young people do. Their vital organ function may decline gradually [1, 2]. Injuries in such patients are usually associated with higher morbidity and mortality [3]. Therefore, improving the efficiency of treatment in elderly patients with trauma is crucial [2, 4].

Chest trauma is common in elderly patients, and posttrauma complications are usually more frequent in older patients than in younger patients. Osteoporosis leads to frequent rib fractures in older people [5]. Conservative treatment combined with bed rest, continuous oxygen therapy, and adequate pain control are usually the first treatment choices for blunt chest trauma in elderly patients [6]. Some patients may develop hemothorax or pneumothorax [7, 8]. However, most cases of posttrauma hemothorax involve a small volume that can be absorbed spontaneously. Once the hemothorax is sufficiently large to compress the lung parenchyma, drainage should be performed. Tube thoracostomy is the most useful tool for managing pleural fluid collections [7], and the procedure can be performed rapidly in emergency department (ED) because it is easy and safe. However, postprocedural complications, such as wound pain, infection, and malpositioned chest tubes, have been reported [9, 10]. Moreover, if the tube is obstructed, hemothorax is retained and the infection rate increases [11, 12]. Video-assisted thoracoscopic surgery (VATS) is currently considered the most effective tool for managing retained pleural collections from chest trauma [13–16].

Although the traditional management of traumatic hemothorax or pneumothorax is by tube thoracostomy, most injured elderly patients present with stable vital signs at our hospital. Therefore, we attempted to reduce the frequency of invasive procedures for these patients to improve the efficiency of hemothorax treatment. VATS is directly performed without an initial tube thoracostomy in some elderly patients. In this study, we assumed that replacing tube thoracostomy with direct VATS would be a more efficient approach to treating hemothorax and improving overall clinical outcomes.

## 2. Materials and Methods

### 2.1. Setting and Patients

This study was conducted in a level-1 trauma medical center in Southern Taiwan. The center has 1300 beds with approximately 1200 emergent traumatic visits per month. Patients aged >65 years with blunt chest trauma who were admitted to the trauma unit at our center were included. In the trauma bay, patients underwent a primary survey according to the Advanced Trauma Life Support (ATLS) guidelines. Patients with chest trauma having stable vital signs received a secondary survey, including chest computed tomography (CT). All elderly patients with more than 300 mL (estimated by CT) of hemothorax for which tube thoracostomy is indicated were included. In these patients, some patients had associated injuries that required emergency operations and tube thoracostomy was not performed in the trauma bay. VATS was arranged to coincide with the operation for the associated injury. These patients were categorized as group 1. Group 2 included the other patients who received an initial tube thoracostomy in the trauma bay. They were admitted to the intensive care unit (ICU) for further care and observation. This study was approved by the ethics committee of the medical center in which this study was conducted.

**Figure 1:**
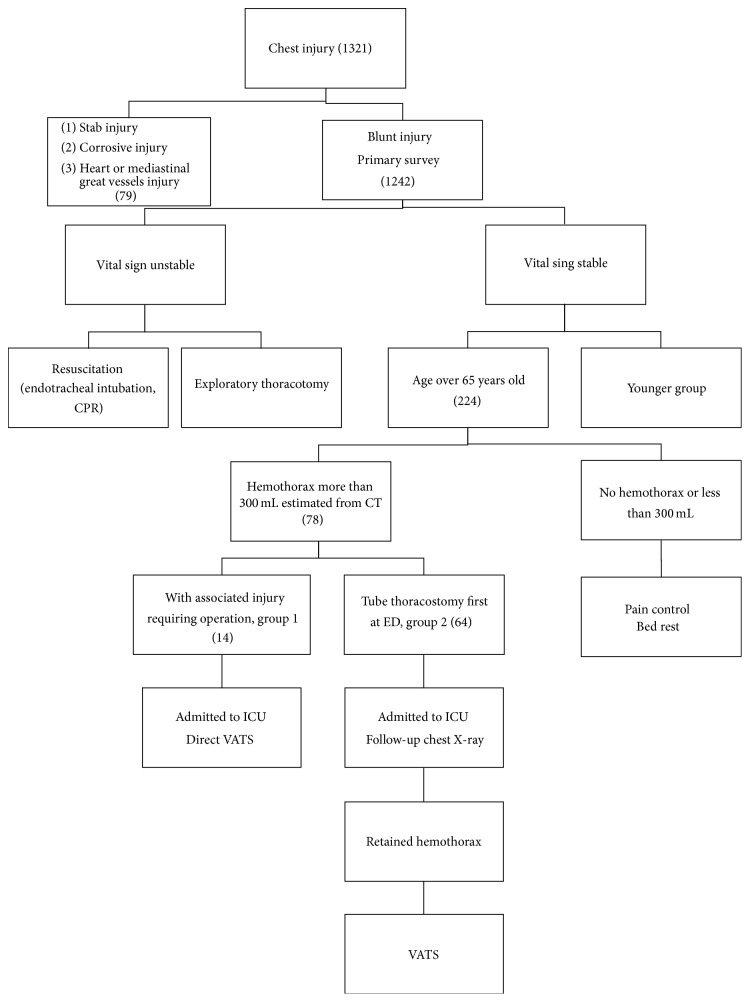
Flowchart of patient collection.

Patients were excluded if they presented with the following: respiratory distress caused by acute airway obstruction or severe lung injuries that required emergent intubation; tension pneumothorax or massive hemothorax with hemodynamic instability requiring emergent tube thoracostomy; more than 1500 mL of blood output upon initial tube thoracostomy placement; ongoing blood loss of >250 mL/h following an emergent thoracotomy; and severe disorders such as liver cirrhosis, chronic obstructive pulmonary disease, chronic renal disease under hemodialysis, and chronic heart failure (i.e., conditions that increase the number of posttrauma complications). The associated injuries were categorized according to the 2005 anatomic injury score (AIS). Patients with an AIS of >3 for one of the associated injuries were excluded.  Figure 1 illustrates the flowchart of patient collection.

After admission to the ICU, both groups were closely monitored for their vital signs. Chest X-rays were obtained daily. Some patients experienced acute respiratory failure because of gradually worsening lung function. In these patients, an endotracheal tube was inserted immediately with positive pressure ventilation. In group 2, a secondary chest CT without contrast was performed in cases where a chest X-ray revealed an increase in the retained pleural collections. Patients were indicated for VATS when a secondary CT displayed retained more than 300 mL of hemothorax.

Patient data included demographic information, the injury mechanism, number of rib fractures, associated injuries, injury severity score (ISS), concomitant chest injuries, postoperative complications, respiratory failure status, number of ventilator days, ICU length of stay (LOS), and in-hospital LOS. Furthermore, the total expense for each patient was evaluated.

All patients received VATS by thoracic surgeons. The same procedures were used for both groups. VATS was performed in an operating room under general anesthesia in the lateral position on the unaffected side, and all patients received prophylactic antibiotics before surgical intervention. A double lumen endobronchial tube was used for a single lung procedure, which provided a clear view for assessing the chest and its contents. Two ports were used during the VATS procedure to connect the camera, evacuate the collections, remove any clots, decorticate the parietal pleura, repair lung injuries, and irrigate the thoracic cavity with normal saline solutions. The procedure was completed when all clots were removed and damaged lung tissues were repaired. Subsequently, the lung was reexpanded. Finally, 2 drainage tubes were fixed into the pleural cavity, and the patient was transferred to the trauma ICU for further postoperative care [17, 18]. Following either procedure, the thoracostomy tubes were removed at the discretion of the thoracic surgeon when the drainage was <100 mL/24 h and no air leak was present.

Posttrauma complications, including the development of empyema, pneumonia, and sepsis, were recorded. Postoperative outcomes were indicated by hospital mortality and the number of ventilator days, as well as ICU and in-hospital LOS. Empyema was defined as pus accumulation in the pleural space (confirmed by bacteria cultures). Pneumonia was defined as an infective condition with bacteria cultures isolated from the sputum after trauma and ventilator use. Sepsis was defined as a systemic response to lung infection with bacteremia.

### 2.2. Statistical Analysis

Simple means were used for obtaining the frequency and percentages of the categorical variables, whereas SDs were used for continuous variables. Categorical variables were compared using the chi-square test or Fisher exact test, and numerical variables were compared using *t*-test or Wilcoxon rank sum test. A *P* value of <0.05 was considered statistically significant. Data analysis was performed using SPSS (Version 19; SPSS Inc., Chicago).

## 3. Results

The study period was from April 2009 to December 2013. We enrolled 1321 patients with chest injury admitted at our hospital, 1242 of whom had blunt chest trauma. Among these patients, 224 patients were aged >65 years (18.0%). Seventy-eight patients had more than 300 mL of hemothorax, indicating that a tube thoracostomy was necessary. All patients had stable vital signs during their stay in the ED. Eighty percent of the participants were men. The mean age was 75 years (SD = 7.59). Half of the cases were motorcycle-related injuries. Eighteen patients (23%) had slipped and fallen while walking. Most of these patients had multiple injuries, and only 14 patients had only one chest trauma.  Table 1 lists the basic demographic data of the patients.

To manage posttraumatic hemothorax, the patients were divided into two groups. Fourteen patients had associated injuries at other anatomical regions for which emergency operations were indicated; tube thoracostomies were not performed in these patients. Direct VATS was arranged synchronously with the associated operations within 2 or 3 days after trauma (group 1). The remaining 64 patients were treated with initial tube thoracostomies in the ED (group 2). Both groups were admitted to the ICU for close observation after their survey completion in the ED.  Table 2 shows a comparison of the patient characteristics and demographics between the 2 groups. No statistically significant differences were observed in the mean patient age between the 2 groups (74.79 versus 75.31 y, *P* = 0.81). The percentage of the women in group 1 was higher than that in group 2 (42.9% versus 12.5%, *P* = 0.007). The total number of fractured ribs in both groups was identical (5.57 versus 5.75, *P* = 0.75). The percentage of flail chest incidents did not differ significantly between the 2 groups (35.7% versus 35.9%, *P* = 0.987). The lung contusion score was slightly higher in group 1 than in group 2 (5.0 versus 3.86, *P* = 0.055). Fifty patients had occult pneumothorax. The group 2 patients had a higher percentage of pneumothorax compared with the group 1 patients (28.6% versus 71.9%, *P* = 0.002). Although some differences in the chest injuries were observed between the 2 groups, the chest AISs of the 2 groups were relatively similar (3.21 versus 3.45, *P* = 0.09). However, the distributions of associated injuries between the 2 groups differed. Group 1 had a higher percentage of abdominal injury than group 2 did (0.79 versus 0.22, *P* = 0.054). Nearly 80% (11/14) of the patients in group 1 had an extremity injury that required surgical intervention. The AIS of the limbs was considerably higher in group 1 than in group 2 (2.14 versus 1.19, *P* = 0.001). The overall ISS between the 2 groups did not differ significantly (18.43 versus 18.38, *P* = 0.974).

Some of the patients in this study developed respiratory distress after being admitted to the ICU. Among these patients, 15 patients (2 in group 1 and 13 in group 2) experienced acute respiratory failure and required endotracheal intubation with ventilator support (14.3% versus 20.3%, *P* = 0.604). The 2 patients in group 1 had occult pneumothorax. Intubations with positive pressure ventilation were applied, and hence the pneumothorax did not progress and a tube thoracostomy was not required. All patients in group 1 received VATS directly. Therefore, VATS was quicker in group 1 than in group 2. Most of them (11/14) received VATS within 4 days after trauma (mean = 3.57 d, SD = 0.94). Nearly 50% of the patients in group 2 (30/64) had retained hemothorax and required VATS. Because the observation time in group 2 was longer than that in group 1, the time from trauma to operation was significantly longer (mean 5.97 d, SD = 3.43, *P* = 0.001).

Table 3 shows a comparison of clinical outcomes between the 2 groups. Microbial cultures from sputum and pleural effusion were collected when the patients had a fever. Group 2 had slightly higher rates of positive microbial cultures from sputum than did group 1 (28.6% versus 56.3%, *P* = 0.061). Positive microbial culture rates from pleural effusion did not differ significantly between the 2 groups (14.3% versus 21.9%, *P* = 0.524). Because of lower pneumonia rates, greater improvement in clinical outcomes was observed in group 1 than in group 2. Total ventilator-dependent periods were shorter in group 1 than in group 2 (4.68 versus 10.03 d, *P* = 0.034), and most of the patients were weaned off the ventilator within 4 days after the operation (mean = 3.54 d, SD = 4.54). The ICU and in-hospital LOS were significantly shorter in group 1 than in group 2 (6.36 versus 9.34 d, *P* = 0.029 and 15.93 versus 23.17 d, *P* = 0.01, resp.). The total expense for group 1 was slightly less than that in group 2, although the difference was nonsignificant (NT$157 781.05 versus NT$213 209.89, *P* = 0.063).

No complication was observed after VATS in either group. All patients in group 1 survived until discharge from the hospital. Four patients in group 2 had severe infection and eventually died before being discharged from the hospital. The difference in overall mortality between the 2 groups was nonsignificant (0% versus 6.3%, *P* = 0.337).

## 4. Discussion

With advancements in medical treatment, population aging is becoming a global problem in developed countries. Traumatic accidents are a risk for older adults, and this risk increases as they become more fragile with age. How to treat these injuries without causing further harm is a critical concern [2, 4]. Most physicians may choose simple and conservative management plans for elderly patients requiring treatment for trauma. Bed rest with oxygen therapy and adequate pain control are usually the first line of treatment in older patients with a chest injury. Invasive interventions are always considered final options. However, recent studies have reported that aggressive resuscitation and intervention benefitted more than 80% of patients in returning to their preexisting level of independent living [1, 3]. To reduce the total in-hospital LOS and medical expenses, prompt aggressive interventions are crucial for older patients.

Reducing the in-hospital LOS is the main goal of managing trauma patients. Posttraumatic infection is a critical reason for prolonging the treatment course. In this study, the group 1 patients had lower rate of pneumonia. We found that, in blunt chest trauma, pulmonary contusion is common. Because alveolar hemorrhages accompanied with pleural collection cause the lung parenchyma to collapse, the rate of posttrauma infection increases. Once posttraumatic pneumonia occurs, antibiotics should be used for a long period. In group 1, early direct VATS made the hemothorax be removed earlier than in group 2. During VATS, the lung is fully inflated under intubation when the patient receives general anesthesia. Collapsed or obstructed lung alveoli can be reopened. Both procedures might prevent infection in the early stage after trauma. In group 2, late VATS enables evacuation of hemothorax only. Most of these patients had contracted pneumonia before VATS. When the antibiotic treatment time is long, the whole hospitalization course is prolonged. In our study, the infection rates from pleural effusion were identical in both groups. However, this result is in contrast to the results of other studies [9, 10]. In our hospital, all ED thoracostomy procedures are supervised by a senior trauma physician. We suggest that under thorough and detailed aseptic procedures, the rate of infection from tube thoracostomies performed in the ED is equal to those performed in an operating room.

The duration from trauma to VATS also influenced the clinical outcomes of patients with blunt chest trauma [19–21]. In this study, the clinical outcomes improved more in group 1 than in group 2. Because tube thoracostomy in the ED was not performed for the patients in group 1, the duration from trauma to VATS was short. Most group 1 patients received treatment for pneumothorax or hemothorax within 72 hours. Under the actual visibility provided by VATS, the pleural collections can be evacuated adequately and precisely. In group 2, it takes at least 2 days to determine whether retained hemothorax has developed after trauma, even when a tube thoracostomy has been performed. Adding the observation time for other associated injuries, the total duration from trauma to VATS can be up to 6 days after trauma. Accompanied with higher rates of posttraumatic pneumonia in group 2, the total in-hospital LOS is increased. In group 2, the tubes were malpositioned in approximately 40% (26/64) of tube thoracostomies performed in the ED. Although the drainage function of most of these chest tubes was satisfactory, retained hemothorax was observed in nearly 50% (30/64) of the patients. This percentage is higher than that reported in other studies, in which retained pleural collections were performed in patients from all age groups (15%–30%) [7, 13, 15]. Therefore, we concluded that retained hemothorax is more common in elderly patients than in younger patients.

When treating pneumothorax, most physicians are concerned that the pneumothorax will progress to tension pneumothorax. Therefore, compared with other patients, those with pneumothorax are more likely to receive chest intubation in the ED. However, Moore et al. [22] reported a low probability of occult pneumothorax progressing, even under positive pressure ventilation. In our study, the 2 patients with occult pneumothorax in group 1 had acute respiratory failure. Both of them received endotracheal intubation with positive pressure ventilation. The pneumothoraces did not progress and VATS was performed smoothly without any postoperative complication. In addition, high pneumothorax rates increased the average chest AIS of group 2. However, the pulmonary contusion score was higher in group 1 than in group 2, thus increasing the average chest AIS. These 2 factors explain the nonsignificant difference in the average chest AIS between the 2 groups.

Previous studies have indicated that associated injuries were the most crucial factors influencing treatment decisions [13, 14]. In group 1 in the present study, 10 patients had bone fractures of the limbs, and one patient sustained a soft tissue crushing injury to the limbs. The planned surgeries were arranged 2-3 days after trauma. Three patients had concomitant intra-abdominal injury. Laparoscopic examinations were arranged because bowel perforation or bleeding from mesenteric tearing was highly suspected. During these limb or abdominal surgical interventions, VATS was performed concurrently. All patients required general anesthesia only once, and the total surgical time was only an additional 30 minutes. Among the 30 patients who received VATS in group 2, 12 patients had associated injuries that required subsequent surgery. These patients received general anesthesia twice. Both the risks associated with anesthesia and the total costs increased.

Fall-related injury is the most common type of trauma among elderly adults because they tend to have an unstable gait [4]. However, in our study, motorcycle accidents were the main reason for chest trauma. Slipping was the second leading cause of geriatric trauma. Even a minor fall can cause severe chest trauma in elderly patients with osteoporosis. In addition, multiple associated injuries have been reported in such patients. Therefore, additional and repeated whole-body surveys are required for these elderly patients.

In addition to decreasing the total in-hospital LOS, direct VATS can also reduce the total expense of hospitalization. The overall expense depends on the in-hospital and ICU LOS, duration of ventilator use, operation and anesthesia times, and total drug costs. The infection rate and in-hospital LOS were higher in group 2 than in group 1. However, more than half of group 2 patients received only tube thoracostomy without VATS because there was no retained hemothorax in these cases. Therefore, the LOS and average total expense were lowered in group 2 by these patients. By contrast, no observation time was required for the tube thoracotomies in the group 1 patients, and this may have reduced their in-hospital LOS. Direct VATS provides excellent and adequate drainage. All of these advantages reduce the infection and in-hospital LOS, thus reducing the costs, because the total expense is mainly influenced by the infection rate and in-hospital LOS. Although the operation costs in group 1 were higher than those in group 2, which was due to a combination of associated surgeries, the average total expense was low. Although no statistically significant difference was observed between the 2 groups in this study, group 1 tended to have lower total expenses than did group 2.

Our study has several limitations. First, patient selections were made subjectively by trauma physicians. Although the treatment guidelines are identical and all trauma surgeons or emergency physicians have received similar ATLS training, the initial treatment for blunt chest trauma cases might differ. Factors such as sex, associated injury, trauma mechanism, and age can influence such decisions. In our hospital, elderly patients usually are initially treated using conservative methods. Second, direct VATS without previous tube thoracostomy is not currently a routine procedure. We use this method only in patients with an associated injury for which emergency surgery has been scheduled. Few patients are suitable for direct VATS; therefore, the total number of cases is small. Third, patients with trauma have a high risk of progressing to more severe conditions. Patients who receive direct VATS should be admitted to the ICU for close observation. All 14 patients in this study had stable blood pressure and the whole course of VATS proceeded smoothly.

## 5. Conclusion

In elderly patients with traumatic hemothorax who present with stable vital signs—particularly those indicated for injury-associated emergency surgery—direct VATS can be performed. The efficiency of the procedure was revealed by lower infection rates and shorter hospitalization stays for patients who received direct VATS than those who did not receive direct VATS.

## Figures and Tables

**Table 1:** Demographic analysis of elderly patients with blunt chest trauma (*n* = 78).

Mean age (yr.), SD	75.22, SD = 7.61
% males	64 (82.1%)
Mechanisms of injury	
Motorcyclist	45
Vehicle driver or passenger	3
Fall accident	16
Cyclist or pedestrian	12
Abuse	2
Anatomic injury score (AIS) of thoracic injury	3.41, SD = 0.57
Associated injuries	
% head injury	36 (46.2%)
% abdominal injury	14 (17.9%)
% extremity injury	55 (70.5%)
Multiple trauma	64 (82.1%)
Median ISS	18.38, SD = 7.57
ICU length of stay (days)	8.81, SD = 7.68
In-hospital length of stay (days)	21.87, SD = 16.03
Expense (new Taiwan dollars, NTD)	177,204.41, SD = 142,656.56
Mortality	4 (5.1%)

ISS: injury severity score; VATS: video-assisted thoracoscopy.

**Table 2:** Comparison of patient characteristics and demographics between the 2 groups.

	Direct VATS without tube thoracostomy in ED (14) group 1	Tube thoracostomy in ED (64) group 2	*P*
Age	74.79, SD = 7.14	75.31, SD = 7.76	0.432
Gender (male)	8 (57.1%)	56 (87.5%)	0.007
Number of fractured ribs	5.57, SD = 1.65	5.75, SD = 2.78	0.753
Flail chest	5 (35.7%)	23 (35.9%)	0.987
Pulmonary contusion score	5.00, SD = 1.92	3.86, SD = 1.72	0.055
Acute respiratory failure in 48 hours after trauma	2 (14.3%)	13 (20.3%)	0.604
Concomitant with pneumothorax	4 (28.6%)	46 (71.9%)	0.002
AIS chest	3.21, SD = 0.43	3.45, SD = 0.59	0.090
AIS head	0.71, SD = 1.07	1.08, SD = 1.40	0.288
AIS abdomen	0.79, SD = 0.98	0.22, SD = 0.63	0.054
AIS extremity	2.14, SD = 0.77	1.19, SD = 0.99	0.001
ISS	18.43, SD = 4.85	18.38, SD = 8.07	0.974
Receiving VATS	14 (100%)	30 (46.9%)	
Time from trauma to VATS (days)	3.57, SD = 0.94	5.97, SD = 3.43	0.001

**Table 3:** Comparison of clinical outcomes between the 2 groups.

	Direct VATS without tube thoracostomy in ED (14) group 1	Tube thoracostomy in ED (64) group 2	*P*
Duration of ventilator support (days)	4.68, SD = 5.65	10.03, SD = 14.81	0.034
Duration of chest tube use	9.36, SD = 3.18	13.40, SD = 8.43	0.004
Positive microbial cultures in sputum	4 (28.6%)	36 (56.3%)	0.061
Positive microbial cultures in pleural effusions	2 (14.3%)	14 (21.9%)	0.524
ICU LOS	6.36, SD = 3.13	9.34, SD = 8.27	0.029
In-hospital LOS	15.93, SD = 6.33	23.17, SD = 17.21	0.010
Expense (NTD)	136747.67, SD = 54495.06	199680.38, SD = 171032.54	0.165
Mortality	0	4 (6.3%)	0.337
